# Dissecting the causal role of immunophenotypes in brain meningioma risk: A Mendelian randomization study

**DOI:** 10.1097/MD.0000000000042678

**Published:** 2025-05-30

**Authors:** Xiaoyang Zhu, Yan Wang, Zhiyuan Liu, Xin Zhang, Shuaiqi Zhang, Bo Pang, Shu Zhu

**Affiliations:** a Binzhou Medicine University, Yantai, Shandong Province, China; b Qingdao Hiser Hospital Affiliated of Qingdao University (Qingdao Traditional Chinese Medicine Hospital), Qingdao, Shandong Province, China.

**Keywords:** cancer prevalence, cancer prevalence and etiology, GWAS, immunophenotypes, Mendelian Randomization, meningoma

## Abstract

Meningiomas are primarily benign brain tumors that, despite their typically slow growth, often cause significant health issues due to their location. Emerging immunotherapy approaches have sparked optimism regarding treatment outcomes for patients. We conducted a comprehensive Mendelian randomization (MR) analysis, utilizing 731 immune cell phenotypes to explore causal relationships with Brain Meningioma. We employed inverse variance weighted as the primary analysis method, MR-Egger and Maximum likelihood as secondary methods, and Weighted median, Weighted mode, and Simple mode as supplementary methods to ascertain causal links. Additionally, we performed sensitivity tests including heterogeneity tests, pleiotropy tests, reverse MR, leave-one-out analysis, and MR-PRESSO to ensure result robustness. Our study identified potential causal relationships of 7 immune phenotypes with Brain Meningioma, comprising 2 risk factors and 5 protective factors. Specifically, B cell-related phenotypes included CD20 on IgD − CD24 − B cell (*P* = .048, OR = 1.094, 95% CI = 1.001 − 1.196), CD38 on IgD − CD38 + B cell (*P* = .004, OR = 0.949, 95% CI = 0.915 − 0.984), and CD38 on IgD − CD38dim B cell (*P* = .02, OR = 1.073, 95% CI = 1.011 − 1.140). T cell-related phenotypes included Activated CD4 regulatory T cell %CD4 + T cell (*P* = .013, OR = 0.885, 95% CI = 0.804 − 0.974) and CD25++ CD45RA − CD4 not regulatory T cell %CD4 + T cell (*P* = .004, OR = 0.905, 95% CI = 0.846 − 0.969). Monocyte-related phenotypes included CD39 on monocyte (*P* = .022, OR = 0.948, 95% CI = 0.905 − 0.992), and dendritic cell-related phenotype included CD86 + myeloid dendritic cell absolute count (*P* = .01, OR = 0.914, 95% CI = 0.853 − 0.979).

## 1. Introduction

Meningioma is a predominantly benign brain tumor that, despite its generally slow growth, can cause significant health problems due to its location.^[1]^ It is one of the most common primary brain tumors in the United States, with approximately 42,060 new cases estimated in 2022 and around 43,170 new cases projected in 2023.^[2]^ Meningiomas are particularly prevalent among all nonmalignant tumors. These tumors primarily cause health issues by exerting pressure on surrounding structures, increasing intracranial pressure, and leading to complications such as brain edema, brainstem compression, and the compression of critical brain vasculature.^[3]^ Consequently, understanding the underlying mechanisms of meningioma is essential.

Recent studies suggest that the immune system may play a significant pro-tumorigenic role in various stages of meningioma development. Genetic variations related to immune system function or response may influence individual susceptibility to meningiomas.^[4,5]^ Furthermore, immune cells might contribute to the risk of meningioma initiation and progression by producing inflammatory mediators or causing tissue damage through inflammatory responses.^[6–8]^ Similarly, during treatment, immune cells may release damage-associated molecular patterns, such as ATP and HMGB1, which stimulate the production of immune-stimulatory cytokines like IL-1α. This response could either trigger a novel antitumor T cell reaction or induce an immunosuppressive response, potentially promoting meningioma growth.^[9,10]^ Additionally, upon recognizing dying meningioma cells, immune cells might release growth factors and extracellular antiapoptotic or anti-cell death signals, such as TNF, EGF, IL-6, and Wnt ligands, which could undermine therapeutic efficacy.^[11]^ However, the current evidence primarily comes from observational studies, which may be subject to confounding factors and reverse causation, with few assessments of the causal relationship between immune cells and meningioma.

Mendelian randomization (MR) is an emerging epidemiological method used to determine whether the associations observed between risk factors and outcomes have a causal effect. It relies on the random assignment of alleles at conception, thereby avoiding confounding factors and reverse causation, and is often referred to as a natural randomized controlled trial. MR uses instrumental variables (IVs) to further analyze causal relationships within a sample. IVs in MR must meet 3 critical assumptions: (1) the relevance assumption, which requires a strong association between the IVs and the exposure; (2) the exclusion restriction assumption, which posits that the IVs is not related to the outcome except through the exposure; and (3) the independence assumption, which requires that the IVs is not associated with confounding factors.

## 2. Method

### 2.1. Experimental design

**Figure 1.**
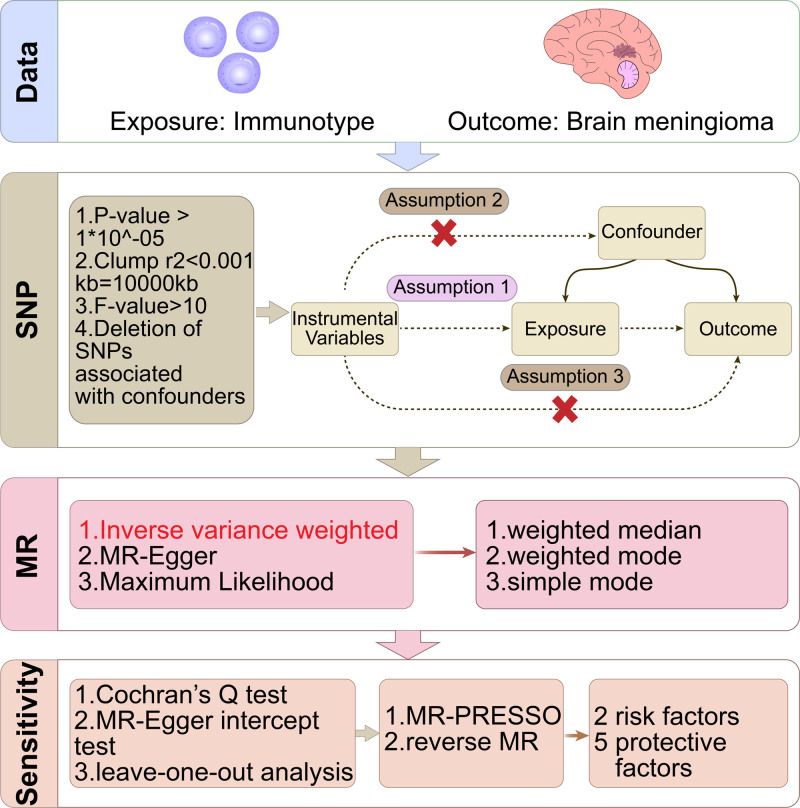
The figure shows the flowchart of the experiment in this article and the principle of Mendelian randomization.

We implemented a two-sample MR to investigate the causal relationship between the immune phenotype and meningioma, as detailed in the figure below. The genome-wide association study (GWAS) data used in this study are derived from previously published research, the GWAS data used in this study are derived from previously published research, and detailed information along with the download link for the data can be found in Table S1, Supplemental Digital Content, https://links.lww.com/MD/P106. The original GWAS referenced in this study has been ethically reviewed and approved by the relevant institutional review boards; therefore, no additional ethical approval or participant consent was required for this research. See Figure 1 for details.

### 2.2. Data sources

The study utilizes publicly available GWAS datasets. Employing immune phenotypes as exposures, derived from data on 3757 Sardinians of European descent collected by Orrù V et al.^[12]^ These datasets are accessible through the GWAS Catalog under accession numbers GCST0001391 to GCST0002121.This dataset includes 731 immune phenotypes of 22 immune cell types, derived from peripheral blood samples through flow cytometry.^[12]^ The outcome data were the summary GWAS data on meningioma, collected and organized by the Finnish database, version R10.^[13]^ The sample included 314,708 individuals of European descent, divided into 1316 cases and 313,392 controls. Meningioma was classified according to the International Classification of Diseases, 10th Revision codes from participants’ hospital records.

### 2.3. IVs selection

Firstly, we selected single nucleotide polymorphism (SNP) with a *P*-value <1 × 10^−5^ to obtain IVs associated with the exposures. Secondly, the criteria of *R*^2^ < 0.001 and kb = 1 0,000 were used to continue screening SNPs to avoid potential bias caused by linkage disequilibrium. Thirdly, all weak IVs with an F-statistic (*F*) <10 were removed to avoid weak instrument bias. The calculation formula for this is *F* = *R*²(*N* − 2)/(1 − *R*²).^[14]^ Additionally, we retrieved information on all IVs used in this study from the Ensembl database and excluded SNPs associated with confounding factors such as sex hormones.

### 2.4. Implementation of MR

This study employed 6 MR methods for analysis. Among these, the inverse variance weighted (IVW) method^[15]^ is the most commonly used primary approach in MR studies. Assuming no horizontal pleiotropy, the IVW results would be unbiased. Therefore, IVW was used as the primary method of analysis in this study. The MR-Egger method offers greater flexibility in addressing situations where genetic variants may affect multiple factors, allowing for a more comprehensive assessment of result reliability. It is suitable for analyses where confounding factors are difficult to control.^[16]^ The Maximum Likelihood method is similar to IVW but takes into account the uncertainty in the association between IVs and exposure.^[17]^ Given the complex etiology of meningioma and the substantial number of immune phenotype IVs, MR-Egger, and Maximum Likelihood were used as secondary methods. Weighted Median, Weighted Mode, and Simple Mode served as supplementary methods. To be considered a positive result, IVW and Maximum Likelihood, and MR-EGGER must simultaneously yield *P*-values <.05, and the effect directions across all 6 methods must be consistent.

### 2.5. Sensitivity analysis

To ensure robustness, a leave-one-out analysis was performed by sequentially removing each SNPs and conducting MR analysis with the remaining SNPs. MR-Egger was used to test for pleiotropy; results with a *P*-value >.05 for the intercept indicated no pleiotropy, thus confirming result stability. Heterogeneity was assessed using Cochran Q test; a *P*-value >.05 suggested no heterogeneity in the outcomes. Additionally, MR-PRESSO was employed to identify outliers and test for pleiotropy. The results of the MR-PRESSO test for pleiotropy are not considered reliable if outlier SNPs are detected. In such cases, the outlier SNPs are removed, and the MR analysis is repeated. The MR-PRESSO test is then conducted again to assess pleiotropy and the presence of outlier SNPs, continuing this process until no outlier SNPs remain. After selecting IVs using the same *P*-value threshold, clumping parameters, and F criteria as in the forward analysis, reverse MR is performed to check for reverse causality in positive results. If reverse causality is detected, the positive result is deemed unreliable.

All analyses were performed using the TwoSampleMR (version 0.6.3) and MR-PRESSO packages in R (version 4.3.3).

## 3. Results

Following IVs selection, a total of 17,528 SNPs from 729 immune phenotypes were identified, all with F exceeding 10 and a mean of approximately 33.45, thus mitigating bias from weak IVs. A total of 8 SNPs associated with sex hormones were identified and excluded from the analysis using the Ensembl database. SNPs as IVs are presented in Table S2, Supplemental Digital Content, https://links.lww.com/MD/P106 and SNPs associated with confounders are presented in Table S3, Supplemental Digital Content, https://links.lww.com/MD/P106.

**Figure 2.**
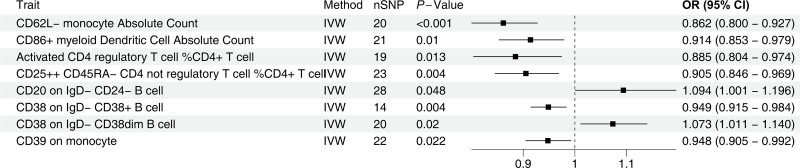
MR results of causal effects between immunotype and meningioma through IVW method. nSNP = number of single nucleotide polymorphism, MR = Mendelian randomization, IVW = inverse variance weighted, OR = odds ratio, CI = confidence interval.

As shown in Figure 2, MR revealed causal relationships between 8immune phenotypes and meningioma, comprising 2 risk factors and 6 protective factors Among these, B cell-related phenotypes included CD20 on IgD − CD24 − B cell (*P* = .048, OR = 1.094, 95% CI = 1.001 − 1.196), CD38 on IgD − CD38 + B cell (*P* = .004, OR = 0.949, 95% CI = 0.915 − 0.984), and CD38 on IgD − CD38dim B cell (*P* = .02, OR = 1.073, 95% CI = 1.011 − 1.140). T cell-related phenotypes comprised Activated CD4 regulatory T cell %CD4 + T cell (*P* = .013, OR = 0.885, 95% CI = 0.804 − 0.974) and CD25++ CD45RA − CD4 not regulatory T cell %CD4 + T cell (*P* = .004, OR = 0.905, 95% CI = 0.846 − 0.969). Monocyte-related phenotypes included CD62L − monocyte absolute count (*P* = .001, OR = 0.862, 95% CI = 0.800 − 0.927) and CD39 on monocyte (*P* = .022, OR = 0.948, 95% CI = 0.905 − 0.992). The dendritic cell (DC)-related phenotype was CD86 + myeloid DC absolute count (*P* = .01, OR = 0.914, 95% CI = 0.853 − 0.979). See Figures S1 and Figure S2, Supplemental Digital Content, https://links.lww.com/MD/P105 for details. The positive results are presented in Table S4, Supplemental Digital Content, https://links.lww.com/MD/P106.

In the sensitivity analysis, no heterogeneity or pleiotropy was observed among all positive results. The MR-PRESSO test indicated the absence of pleiotropy and SNPs outliers. The leave-one-out method confirmed that the results were not driven by any single SNP, demonstrating robustness. See Figure S3, Supplemental Digital Content, https://links.lww.com/MD/P105 for detail. The sensitivity analysis of positive results is presented in Table S5, Supplemental Digital Content, https://links.lww.com/MD/P106.

However, reverse MR identified a potential issue of reverse causality, as the IVW and Maximum likelihood methods for CD62L-monocyte absolute count yielded *P*-values <.05. Consequently, these results were excluded from the analysis. The results of reverse MR for all immune phenotypes identified using forward MR are presented in Table S6, Supplemental Digital Content, https://links.lww.com/MD/P106. To maintain academic transparency, we have placed the negative MR results obtained in this article in Table S7, Supplemental Digital Content, https://links.lww.com/MD/P106.

## 4. Discussion

Our study employed MR analysis to establish causal relationships between 7 immune phenotypes from 4 types of cells and the incidence of meningioma, identifying 2 risk factors and 5 protective factors. Furthermore, sensitivity analysis and implementation of reverse MR ensured the robustness and reliability of our findings. These results contribute to a deeper understanding of meningioma pathogenesis, providing a critical scientific foundation for subsequent clinical interventions and preventive strategies.

The identified immune phenotypes bear the following clinical significance. CD38 is involved in B cell activation, contributing to immunomodulatory effects by reducing the number of regulatory B cells. It also plays a role in cellular adhesion and interactions between B cells and other immune cells.^[16]^ CD38 may serve as an independent adverse prognostic factor in meningioma.^[18]^ CD20 is primarily associated with B cell activation, proliferation, and survival. Clinically, CD20 positivity reflects B cell infiltration and the secretion of IL-10 by tumor-associated macrophages (TAMs) with M2 polarization, which significantly contributes to an immunosuppressive microenvironment.^[19]^ CD4 enhances antigen recognition and response by regulatory T cells through binding to major histocompatibility complex class II molecules. Since Tregs are responsible for maintaining immune tolerance and suppressing excessive immune responses,^[20]^ enhancing CD4 signaling or utilizing CD4 agonists may improve antitumor immunity against meningioma.^[21]^ CD25++ plays a critical role in T cell activation and proliferation and may be associated with tumor cell proliferation and survival capacity. Its expression in meningiomas may serve as a component of diagnostic evaluation.^[22]^ CD45RA⁻ may be involved in the activation of memory-like T cells, thereby reflecting their functional status.^[23]^ CD62L is associated with monocyte adhesion and migration,^[24]^ and may act as a novel biomarker for predicting responses to immunotherapy. CD39 is involved in the regulation of immune responses and inflammation^[25]^; in meningioma, CD39 appears to function as a protective factor, suggesting its potential as a therapeutic target. CD86⁺ DCs can interact with the CD28 receptor on T cells, providing the necessary co-stimulatory signal for T cell activation. Therefore, CD86 may serve as a therapeutic target in immunotherapy by enhancing the antitumor function of DCs through upregulation of CD86 expression.^[26]^

Previous studies may provide insight into the potential mechanisms by which the immune markers we identified either promote or inhibit the pathogenesis of meningioma. The pathogenesis of meningioma involves various factors, including genetic predisposition, the inflammatory microenvironment, immune evasion, hormonal influences from hormone replacement therapy, TERT promoter mutations, and ionizing radiation.^[27–29]^ CD38 contributes to adenosine production within the tumor microenvironment, thereby suppressing the activity of immune cells – particularly natural killer cells and T lymphocytes – and promoting tumor progression.^[16]^ However, CD38 can also facilitate tumor cell death through mechanisms such as antibody-dependent cellular cytotoxicity, antibody-dependent cellular phagocytosis, and complement-dependent cytotoxicity. Moreover, CD38-targeting antibodies have demonstrated immunomodulatory properties, including the suppression of regulatory T cells, thereby enhancing the antitumor immune response.^[16]^ High density of CD20⁺ cells is positively correlated with CD8⁺ T cell infiltration and has been associated with favorable prognosis.^[19]^ CD4 expressed on regulatory T cells enhances antigen recognition and response during activation by interacting with antigen-presenting cells, thereby maintaining immune homeostasis.^[20]^ Activation of CD25 promotes cell proliferation and survival through the IL-2 signaling pathway; additionally, CD25⁺ cells may facilitate immune evasion by tumors through suppression of effector T cell activity.^[22]^ CD62L contributes to antitumor immunity by promoting the migration of CD8⁺ T cells into the tumor microenvironment.^[24]^ CD86 interacts with the CD28 receptor on T cells, thereby enhancing T cell activation and proliferation.^[26]^

Previous research indicates that immune cells can either induce or promote meningiomas. Firstly, the interaction between MIF and CD74 on macrophages serves as a primary pathway for MIF signaling, which is implicated in promoting TAM development. Blocking CD74-positive macrophages from receiving MIF signals can inhibit this process.^[30]^ Additionally, elevated expression of the CDCA7 gene in cells correlates significantly with tumor-infiltrating immune cells and immune checkpoint molecules associated with tumorigenesis, such as CD80, CD276, CD28, PDCD1LG2, and TNFSF4.^[31]^ Moreover, in meningiomas of the frontal cortex, the immune system leads to upregulation of PD-L1 and Lgals9 as immune escape markers, thereby shielding neurotumor cells from infiltrating T cells.^[32]^ Single-cell transcriptomics has identified MES1 and MES2 tumor cells that strongly correlate with immune infiltration, expressing high levels of immune factors like CSF1 and CCL2, shaping an immunosuppressive tumor microenvironment.^[33]^ Innate immune cells produce cytokines and chemokines that attract leukocytes to participate in NFκB activation, a transcription factor closely linked to inflammation and tumor development, promoting uncontrolled cell cycle progression and antiapoptotic capabilities in neurotumor cells.^[34]^

Moreover, previous studies indirectly support our findings of strong causal relationships. DCs are regulated by various signals in the tumor microenvironment, including cellular stress and cell death signals, altering their function towards a pro-tumorigenic direction.^[35]^ For instance, certain tumor cells secrete chemokines like CCL4 and CXCL12 to attract specific subsets of DCs, such as pDCs, to express immune checkpoint molecules PD-L1, ICOSL, and tryptophan 2,3-dioxygenase, thereby dampening their antitumor immune response.^[36,37]^ During tumor progression, DCs may be influenced by hypoxic and acidic microenvironments, exhibiting pro-tumorigenic traits such as inducing regulatory T cells,^[38]^ thus inhibiting effective antitumor immunity. The state of DCs in tumors can be altered by HMGB1 released by tumor cells,^[39,40]^ affecting their maturation and antigen-presenting capabilities, transitioning them into a tolerant state that promotes tumor development. Certain transcription factors and epigenetic mechanisms play crucial roles in the exhaustion and functional decline of T cells, such as the NR4A transcription factor limiting CAR T cell function in solid tumors.^[41–47]^ While tumor-infiltrating B cells are generally viewed as positive contributors to antitumor immunity, they can adopt regulatory phenotypes in some cases, suppressing antitumor immune responses, and antibodies produced by B cells may promote tumor growth under certain conditions, such as by activating complement or macrophages, potentially inducing pro-tumor inflammation.^[48]^ Monocytes can differentiate into TAMs, which typically exhibit pro-tumorigenic properties by secreting pro-angiogenic factors and immune suppressive molecules, exacerbating tumor progression.^[49,50]^ Monocyte-derived suppressor cells express specific surface markers like CD14, CD33, and HLA-DR, inhibiting T cell function and playing an immunosuppressive role in the tumor microenvironment.^[51]^

From a methodological perspective, we consider a result to be positive only when the *P*-values of the IVW, MR-EGGER, and Maximum Likelihood methods are all below .05, and the directions of effect across all 6 methods are consistent. Stephen Burgess et al emphasized that the MR assumption requiring SNPs to be independent of confounding factors must be empirically supported either through scientific understanding or the application of statistical methods. They further noted that for many exposures, the causal relationship between SNPs and exposures cannot be definitively established.^[52]^ In this study, a total of 17,528 SNPs were used as IVs for immune cell phenotypes. Although we rigorously assessed all SNPs for potential confounding factors and conducted stringent weak IVs filtering, we introduced the MR-EGGER and Maximum Likelihood methods as secondary methods to address these challenges. Jack Bowden et al suggested that MR-EGGER, as a statistical method, can be applied when IVs assumptions are violated but weaker assumptions are met.^[16]^ While Maximum Likelihood and IVW are fundamentally similar, Maximum Likelihood places greater emphasis on the uncertainty between IVs and exposures compared to IVW.^[53]^

In summary, our study benefits from employing various MR methods to establish stringent positive criteria as *P*-value thresholds, effectively avoiding type I errors, and mitigating confounding factors and reverse causation effects compared to typical clinical cohort studies. Nevertheless, there are limitations in our experimental design; firstly, the GWAS dataset used was limited to individuals of European ancestry, thus analysis of patients from all ancestries was not conducted. Additionally, the cohort size exposed to immune cell phenotypes in GWAS was relatively small, underscoring the need for appropriate GWAS datasets in future studies. In conclusion, the findings on the immune phenotype of meningioma presented in this study are generally reliable.

Notably, due to the lack of multi-ethnic GWAS data, all participants in this study were exclusively derived from populations of European ancestry. Consequently, caution should be exercised when extrapolating the research findings to other ethnic groups, such as those of Asian or African descent. Further validation is required once genome-wide association studies data from diverse racial populations become available.

## Acknowledgments

We are grateful to all volunteers who participated in this study. We want to acknowledge the participants and investigators of the FinnGen study. Thanks to the Genome-Wide Association Study Alliance for making these summary statistics publicly available. A biostatistician may review such manuscripts during the review process.

## Author contributions

**Funding acquisition:** Shu Zhu.

**Writing – original draft:** Xiaoyang Zhu, Yan Wang, Zhiyuan Liu, Shu Zhu.

**Writing – review & editing:** Xiaoyang Zhu, Yan Wang, Xin Zhang, Shuaiqi Zhang, Bo Pang, Shu Zhu.

## Supplementary Material





## References

[1] Wiemels J Wrensch M Claus EB. Epidemiology and etiology of meningioma. J Neurooncol. 2010;99:307–14. 20821343 10.1007/s11060-010-0386-3 PMC2945461

[2] Ostrom QT Price M Neff C. CBTRUS statistical report: primary brain and other central nervous system tumors diagnosed in the United States in 2016-2020. Neuro Oncol. 2023;25(12 Suppl 2):iv1–iv99. 37793125 10.1093/neuonc/noad149 PMC10550277

[3] Buerki RA Horbinski CM Kruser T Horowitz PM James CD Lukas RV. An overview of meningiomas. Future Oncol. 2018;14:2161–77. 30084265 10.2217/fon-2018-0006 PMC6123887

[4] Rajaraman P Wang SS Rothman N. Polymorphisms in apoptosis and cell cycle control genes and risk of brain tumors in adults. Cancer Epidemiol Biomarkers Prev. 2007;16:1655–61. 17684142 10.1158/1055-9965.EPI-07-0314

[5] Rajaraman P Brenner AV Neta G. Risk of meningioma and common variation in genes related to innate immunity. Cancer Epidemiol Biomarkers Prev. 2010;19:1356–61. 20406964 10.1158/1055-9965.EPI-09-1151 PMC3169167

[6] Trinchieri G. Cancer and inflammation: an old intuition with rapidly evolving new concepts. Annu Rev Immunol. 2012;30:677–706. 22224761 10.1146/annurev-immunol-020711-075008

[7] Rothwell PM Fowkes FG Belch JF Ogawa H Warlow CP Meade TW. Effect of daily aspirin on long-term risk of death due to cancer: analysis of individual patient data from randomised trials. Lancet. 2011;377:31–41. 21144578 10.1016/S0140-6736(10)62110-1

[8] Rothwell PM Wilson M Price JF Belch JF Meade TW Mehta Z. Effect of daily aspirin on risk of cancer metastasis: a study of incident cancers during randomised controlled trials. Lancet. 2012;379:1591–601. 22440947 10.1016/S0140-6736(12)60209-8

[9] Ghiringhelli F Apetoh L Tesniere A. Activation of the NLRP3 inflammasome in dendritic cells induces IL-1beta-dependent adaptive immunity against tumors. Nat Med. 2009;15:1170–8. 19767732 10.1038/nm.2028

[10] Hou J Greten TF Xia Q. Immunosuppressive cell death in cancer. Nat Rev Immunol. 2017;17:401. 10.1038/nri.2017.46 PMC6309557 28480899

[11] Srivatsa S Paul MC Cardone C. EGFR in tumor-associated myeloid cells promotes development of colorectal cancer in mice and associates with outcomes of patients. Gastroenterology. 2017;153:178–90.e10. 28400195 10.1053/j.gastro.2017.03.053 PMC5766132

[12] Orrù V Steri M Sidore C. Complex genetic signatures in immune cells underlie autoimmunity and inform therapy. Nat Genet. 2020;52:1036–45. 32929287 10.1038/s41588-020-0684-4 PMC8517961

[13] Kurki MI Karjalainen J Palta P. FinnGen provides genetic insights from a well-phenotyped isolated population. Nature. 2023;613:508–18. 36653562 10.1038/s41586-022-05473-8 PMC9849126

[14] Burgess S Thompson SG; CRP CHD Genetics Collaboration. Avoiding bias from weak instruments in Mendelian randomization studies. Int J Epidemiol. 2011;40:755–64. 21414999 10.1093/ije/dyr036

[15] Burgess S Scott RA Timpson NJ Davey Smith G Thompson SG; EPIC- InterAct Consortium. Using published data in Mendelian randomization: a blueprint for efficient identification of causal risk factors. Eur J Epidemiol. 2015;30:543–52. 25773750 10.1007/s10654-015-0011-z PMC4516908

[16] Bowden J Davey Smith G Burgess S. Mendelian randomization with invalid instruments: effect estimation and bias detection through Egger regression. Int J Epidemiol. 2015;44:512–25. 26050253 10.1093/ije/dyv080 PMC4469799

[17] Pierce BL Burgess S. Efficient design for Mendelian randomization studies: subsample and 2-sample instrumental variable estimators. Am J Epidemiol. 2013;178:1177–84. 23863760 10.1093/aje/kwt084 PMC3783091

[18] Szlasa W Czarny J Sauer N. Targeting CD38 in neoplasms and non-cancer diseases. Cancers (Basel). 2022;14:4169. 36077708 10.3390/cancers14174169 PMC9454480

[19] Roulleaux Dugage M Nassif EF Italiano A Bahleda R. Improving immunotherapy efficacy in soft-tissue sarcomas: a biomarker driven and histotype tailored review. Front Immunol. 2021;12:775761. 34925348 10.3389/fimmu.2021.775761 PMC8678134

[20] Kim J Li J Wei J Lim SA. Regulatory T cell metabolism: a promising therapeutic target for cancer treatment? Immune Netw. 2025;25:e13. 40078783 10.4110/in.2025.25.e13 PMC11896657

[21] Härm J Fan YT Brenner D. Navigating the metabolic landscape of regulatory T cells: from autoimmune diseases to tumor microenvironments. Curr Opin Immunol. 2025;92:102511. 39674060 10.1016/j.coi.2024.102511

[22] Karube K Sakihama S Takatori M. Recent progress in pathological understanding of adult T-cell leukemia/lymphoma in the new classification era. Leuk Res. 2025;148:107634. 39689447 10.1016/j.leukres.2024.107634

[23] Ono M Satou Y. Spectrum of Treg and self-reactive T cells: single cell perspectives from old friend HTLV-1. Discov Immunol. 2024;3:kyae006. 38863793 10.1093/discim/kyae006 PMC11165433

[24] Ezdoglian A Tsang-A-Sjoe M Khodadust F. Monocyte-related markers as predictors of immune checkpoint inhibitor efficacy and immune-related adverse events: a systematic review and meta-analysis. Cancer Metastasis Rev. 2025;44:35. 39982537 10.1007/s10555-025-10246-6 PMC11845441

[25] Izuka S Komai T Tsuchida Y Tsuchiya H Okamura T Fujio K. The role of monocytes and macrophages in idiopathic inflammatory myopathies: insights into pathogenesis and potential targets. Front Immunol. 2025;16:1567833. 40181992 10.3389/fimmu.2025.1567833 PMC11965591

[26] Wang D Cui Q Yang YJ Liu AQ Zhang G Yu JC. Application of dendritic cells in tumor immunotherapy and progress in the mechanism of anti-tumor effect of Astragalus polysaccharide (APS) modulating dendritic cells: a review. Biomed Pharmacother. 2022;155:113541. 36127221 10.1016/j.biopha.2022.113541

[27] Rowbottom H Šmigoc T Ravnik J. Malignant meningiomas: from diagnostics to treatment. Diagnostics (Basel). 2025;15:538. 40075786 10.3390/diagnostics15050538 PMC11898517

[28] Yuen CA Zheng M Saint-Germain MA Kamson DO. Meningioma: novel diagnostic and therapeutic approaches. Biomedicines. 2025;13:659. 40149634 10.3390/biomedicines13030659 PMC11940373

[29] Nishida A Andoh A. The role of inflammation in cancer: mechanisms of tumor initiation, progression, and metastasis. Cells. 2025;14:488. 40214442 10.3390/cells14070488 PMC11987742

[30] Fan H Song L Fan J. Decoding meningioma heterogeneity and neoplastic cell-macrophage interaction through single-cell transcriptome profiling across pathological grades. J Transl Med. 2023;21:751. 37880655 10.1186/s12967-023-04445-4 PMC10599053

[31] Wang Y Zhao Y Zhang Z. High expression of CDCA7 in the prognosis of glioma and its relationship with ferroptosis and immunity. Genes (Basel). 2023;14:1406. 37510310 10.3390/genes14071406 PMC10380011

[32] Borgenvik A Holmberg KO Bolin S. Dormant SOX9-positive cells facilitate MYC-driven recurrence of medulloblastoma. Cancer Res. 2022;82:4586–603. 36219398 10.1158/0008-5472.CAN-22-2108 PMC9755969

[33] Xiao Y Wang Z Zhao M. Single-cell transcriptomics revealed subtype-specific tumor immune microenvironments in human glioblastomas. Front Immunol. 2022;13:914236. 35669791 10.3389/fimmu.2022.914236 PMC9163377

[34] Markus RP Fernandes PA Kinker GS da Silveira Cruz-Machado S Marçola M. Immune-pineal axis - acute inflammatory responses coordinate melatonin synthesis by pinealocytes and phagocytes. Br J Pharmacol. 2018;175:3239–50. 29105727 10.1111/bph.14083 PMC6057910

[35] Carrega P Bonaccorsi I Di Carlo E. CD56(bright)perforin(low) noncytotoxic human NK cells are abundant in both healthy and neoplastic solid tissues and recirculate to secondary lymphoid organs via afferent lymph. J Immunol. 2014;192:3805–15. 24646734 10.4049/jimmunol.1301889

[36] Spranger S Bao R Gajewski TF. Melanoma-intrinsic β-catenin signalling prevents anti-tumour immunity. Nature. 2015;523:231–5. 25970248 10.1038/nature14404

[37] Gerlini G Urso C Mariotti G. Plasmacytoid dendritic cells represent a major dendritic cell subset in sentinel lymph nodes of melanoma patients and accumulate in metastatic nodes. Clin Immunol. 2007;125:184–93. 17827069 10.1016/j.clim.2007.07.018

[38] Fujita M Scheurer ME Decker SA. Role of type 1 IFNs in antiglioma immunosurveillance--using mouse studies to guide examination of novel prognostic markers in humans. Clin Cancer Res. 2010;16:3409–19. 20472682 10.1158/1078-0432.CCR-10-0644 PMC2896455

[39] Demoulin S Herfs M Somja J Roncarati P Delvenne P Hubert P. HMGB1 secretion during cervical carcinogenesis promotes the acquisition of a tolerogenic functionality by plasmacytoid dendritic cells. Int J Cancer. 2015;137:345–58. 25492101 10.1002/ijc.29389

[40] Chiba S Baghdadi M Akiba H. Tumor-infiltrating DCs suppress nucleic acid-mediated innate immune responses through interactions between the receptor TIM-3 and the alarmin HMGB1. Nat Immunol. 2012;13:832–42. 22842346 10.1038/ni.2376 PMC3622453

[41] Yang R Cheng S Luo N. Distinct epigenetic features of tumor-reactive CD8+ T cells in colorectal cancer patients revealed by genome-wide DNA methylation analysis. Genome Biol. 2019;21:2. 31892342 10.1186/s13059-019-1921-y PMC6937914

[42] Mognol GP Spreafico R Wong V. Exhaustion-associated regulatory regions in CD8(+) tumor-infiltrating T cells. Proc Natl Acad Sci U S A. 2017;114:E2776–85. 28283662 10.1073/pnas.1620498114 PMC5380094

[43] Liu X Wang Y Lu H. Genome-wide analysis identifies NR4A1 as a key mediator of T cell dysfunction. Nature. 2019;567:525–9. 30814730 10.1038/s41586-019-0979-8 PMC6507425

[44] Harant H Lindley IJ. Negative cross-talk between the human orphan nuclear receptor Nur77/NAK-1/TR3 and nuclear factor-kappaB. Nucleic Acids Res. 2004;32:5280–90. 15466594 10.1093/nar/gkh856 PMC521667

[45] Chen J López-Moyado IF Seo H. NR4A transcription factors limit CAR T cell function in solid tumours. Nature. 2019;567:530–4. 30814732 10.1038/s41586-019-0985-x PMC6546093

[46] Odagiu L May J Boulet S Baldwin TA Labrecque N. Role of the orphan nuclear receptor NR4A family in T-cell biology. Front Endocrinol (Lausanne). 2020;11:624122. 33597928 10.3389/fendo.2020.624122 PMC7883379

[47] Tang L Zhang Y Hu Y Mei H. T cell exhaustion and CAR-T immunotherapy in hematological malignancies. Biomed Res Int. 2021;2021:6616391. 33728333 10.1155/2021/6616391 PMC7936901

[48] Engelhard V Conejo-Garcia JR Ahmed R. B cells and cancer. Cancer Cell. 2021;39:1293–6. 34597591 10.1016/j.ccell.2021.09.007

[49] Tiemessen MM Jagger AL Evans HG van Herwijnen MJ John S Taams LS. CD4+CD25+Foxp3+ regulatory T cells induce alternative activation of human monocytes/macrophages. Proc Natl Acad Sci U S A. 2007;104:19446–51. 18042719 10.1073/pnas.0706832104 PMC2148309

[50] Pommier A Audemard A Durand A. Inflammatory monocytes are potent antitumor effectors controlled by regulatory CD4+ T cells. Proc Natl Acad Sci U S A. 2013;110:13085–90. 23878221 10.1073/pnas.1300314110 PMC3740849

[51] Jakubzick CV Randolph GJ Henson PM. Monocyte differentiation and antigen-presenting functions. Nat Rev Immunol. 2017;17:349–62. 28436425 10.1038/nri.2017.28

[52] Burgess S Woolf B Mason AM Ala-Korpela M Gill D. Addressing the credibility crisis in Mendelian randomization. BMC Med. 2024;22:374. 39256834 10.1186/s12916-024-03607-5 PMC11389083

[53] Burgess S Butterworth A Thompson SG. Mendelian randomization analysis with multiple genetic variants using summarized data. Genet Epidemiol. 2013;37:658–65. 24114802 10.1002/gepi.21758 PMC4377079

