# Supplementary material for: Dissecting the causal role of immunophenotypes in brain meningioma risk: A Mendelian randomization study
Source: Medicine (Baltimore). 2025 May 30;104(22):e42678. doi: 10.1097/MD.0000000000042678 (PMC12129537; doi:10.1097/MD.0000000000042678)

Supplementary Figure 1 MR results of causal effects between immunotype and meningioma. Abbreviations: nSNP, number of single nucleotide polymorphism; MR, Mendelian randomization; IVW, Inverse variance weighted; OR, odds ratio; CI, confidence interval

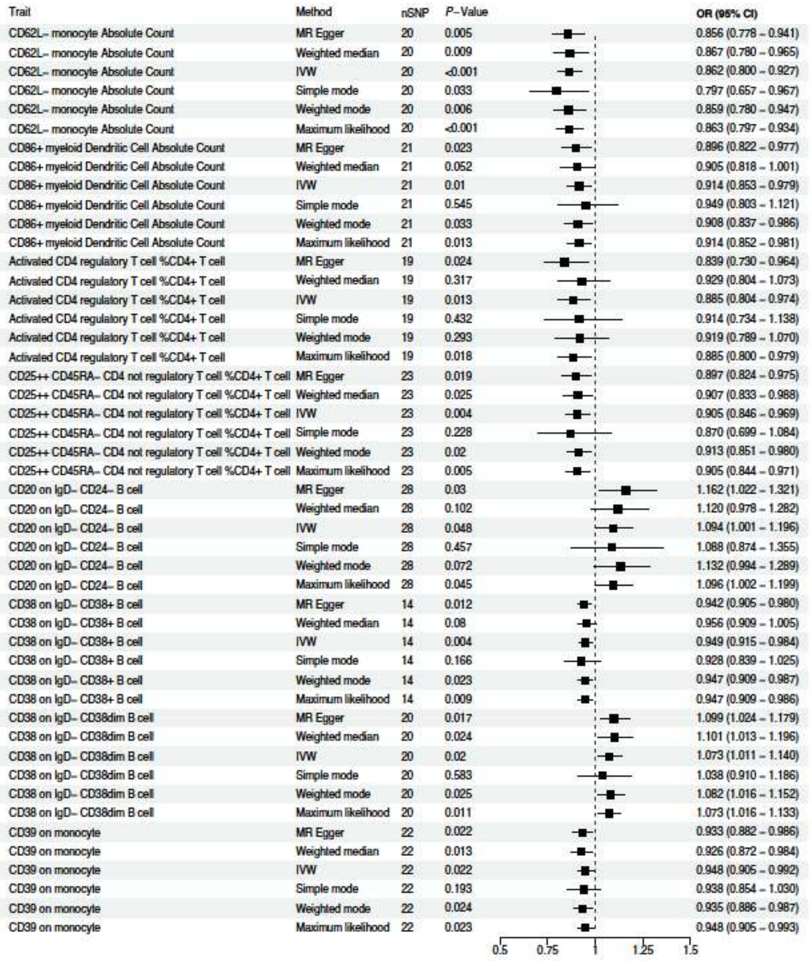

Supplementary Figure 2 Scatter plots of the influence of the significant immunotype on meningioma.

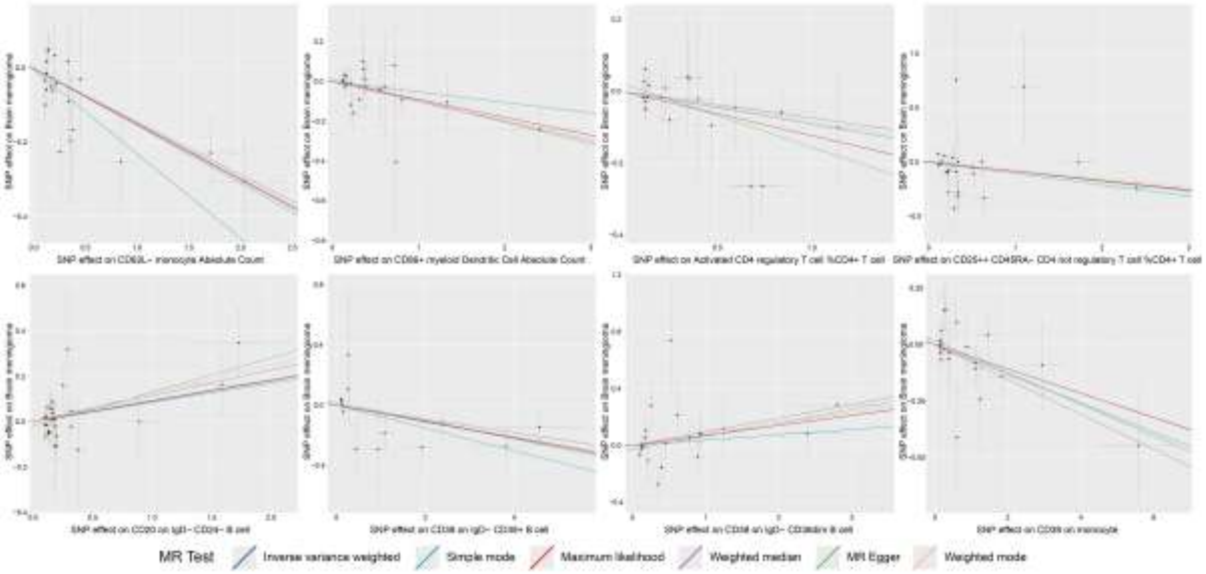

Supplementary Figure 3 Leave-one-out analysis of the causal effects of the significant immunotype on meningioma

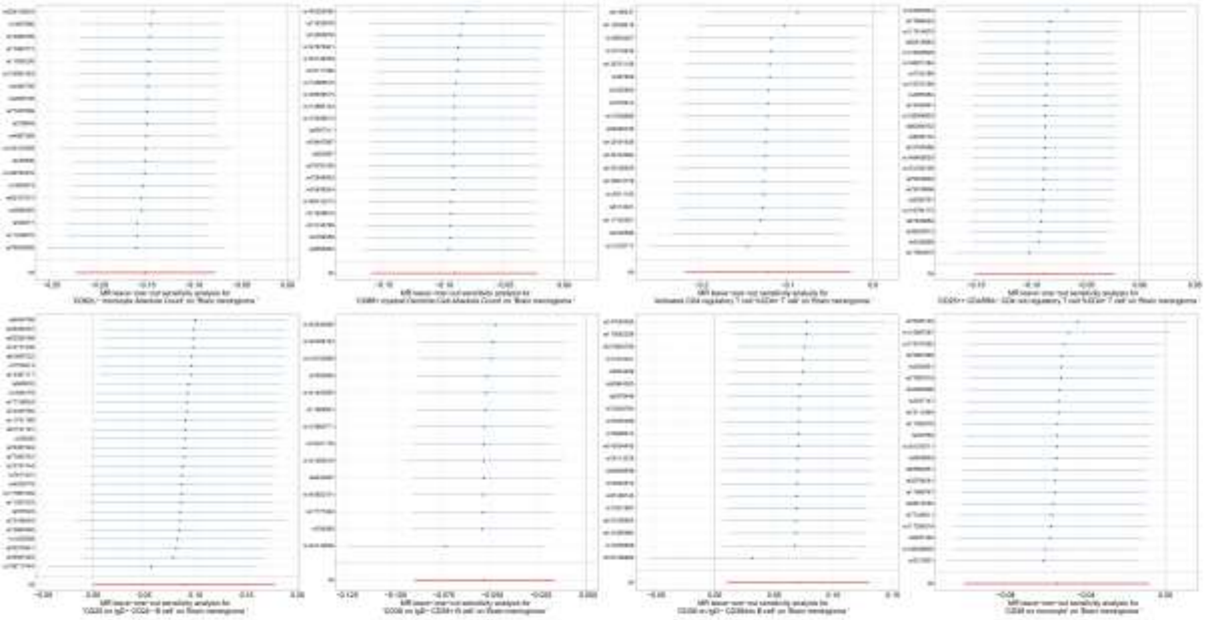

Supplement: Supplementary file 2 [file medi-104-e42678-s002.pdf]
